# Longitudinal Extensive Transverse Myelitis After Respiratory Syncytial Virus Vaccination With Positive Anti-Recoverin Antibodies

**DOI:** 10.1155/crnm/6597450

**Published:** 2025-08-30

**Authors:** Stefania Kalampokini, Ntouigou Fountouktsi, Martha Spilioti, Stefanos Finitsis, Vasilios K. Kimiskidis

**Affiliations:** ^1^First Department of Neurology, AHEPA University Hospital, Aristotle University of Thessaloniki, Stilponos Kyriakidi 1, Thessaloniki, Greece; ^2^Department of Diagnostic and Interventional Neuroradiology, AHEPA University Hospital, Stilponos Kyriakidi 1, Aristotle University of Thessaloniki, Thessaloniki, Greece

## Abstract

Longitudinal extensive transverse myelitis (LETM) is a rare adverse event after vaccination. We present a case of severe myelitis in a 76-year-old man with positive anti-recoverin antibodies that occurred one week after RSVPreF3 vaccination against respiratory syncytial virus (RSV). The patient presented with severe spastic paraparesis, urinary retention, postural tremor of the upper extremities, hypesthesia, severely impaired proprioception and vibration sense in the lower extremities, and tonic spasms of the lower extremities. An MRI of the spine revealed a C3-T9 LETM, with inflammatory cerebrospinal fluid (CSF). The patient was found to have positive anti-recoverin antibodies in serum and CSF. While the patient had an initial improvement on high-dose intravenous steroids, he failed to respond to plasmapheresis. Subsequently, he received intravenous immunoglobulins with mild improvement of his symptoms. The patient's symptoms could be attributed to vaccine-induced inflammatory syndrome. The relationship between anti-recoverin antibodies and central nervous system involvement is likely due to the sharing of epitopes between recoverin and endogenous antigens of the central nervous system. The association between RSV vaccination and LETM has not been previously reported.

## 1. Introduction

Respiratory syncytial virus (RSV) can cause significant morbidity and mortality in older adults and patients with immunosuppression, hematologic malignancies, chronic lung disease, or cardiovascular diseases [1]. Currently, RSV vaccination is recommended for persons aged 60 years or older, in order to prevent severe RSV-associated respiratory infections [1]. The majority of reported adverse events after RSV vaccination were comparable in frequency and intensity to vaccinations against other respiratory viruses and usually resolved within a few days [2]. To date, there have been two cases of acute demyelinating encephalomyelitis (ADEM) and two cases of Guillain–Barré syndrome (GBS), reported within two to 6 weeks of RSV vaccination [2, 3]. In one of these cases, the RSV vaccine was administered three weeks after a COVID-19 mRNA booster vaccine and five days after an influenza vaccine [3]. Here, we present a case of severe longitudinal extensive transverse myelitis (LETM) with positive anti-recoverin antibodies that occurred 1 week after RSVPreF3 vaccination.

## 2. Case Presentation

A 76-year-old man presented to our neurological department due to spastic paraparesis and tonic spasms of the lower extremities over the last two days, which occurred spontaneously or after a tactile stimulus (video (available here)). The patient had also reported urinary retention since the day before and transient diplopia. He had developed a fever up to 38°C a week ago; due to this, he had received moxifloxacin. Before that, he had been vaccinated against RSV with RSVPreF3, an AS01E-adjuvanted recombinant stabilized perfusion F protein vaccine. His past medical history was notable for chronic bronchitis, hyperthyroidism, and a melanoma resection 20 years ago. On examination, the patient was afebrile and had slight psychomotor retardation but was fully oriented and had no meningismus. Muscle strength was significantly reduced in lower limbs with Medical Research Council (MRC) 2/5 proximally and 4/5 distally. Tendon reflexes were brisk in the upper and lower extremities, with extensor plantar response on the left and equivocal response on the right. In addition, there was postural tremor of the upper extremities, hypesthesia and hypalgesia of the lower extremities with a T4 sensory level, abolished vibration sense, and severely impaired proprioception in the lower extremities.

**Figure 1:**
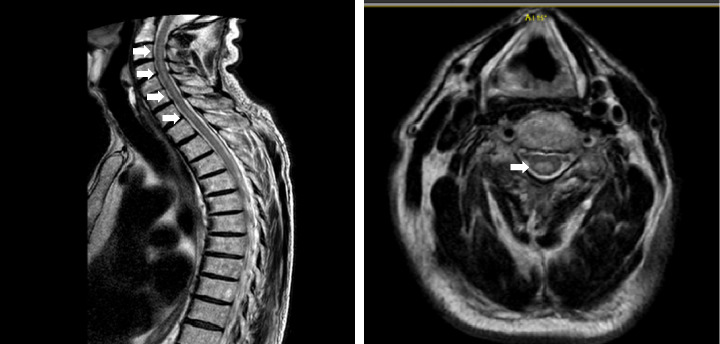
MRI of the spine at presentation (T2 sequence, sagittal and axial), revealing a longitudinal C3-T9 myelitis, shown with white arrows.

**Figure 2:**
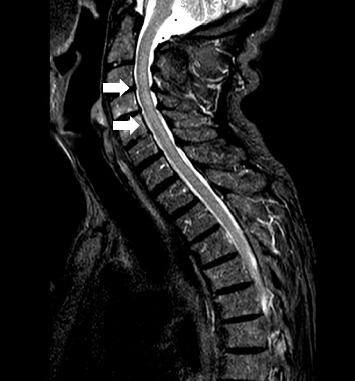
MRI of the spine at 3 weeks (T2 fat-saturated), showing partial resolution of LETM.

A magnetic resonance imaging (MRI) of the spine disclosed a C3-T9 LETM (Figure 1), whereas a brain MRI was unremarkable. Cerebrospinal fluid (CSF) examination showed 50 cells (predominantly lymphocytes, Ref. ≤ 4), glucose of 55 mg/dL (Ref. 50–80 mg/dL), and slightly elevated protein of 67.1 mg/dL (Ref. 20–60 mg/dL). Visual evoked potentials and fundoscopy were normal. The patient was started on acyclovir 750 mg t.i.d., which was discontinued a week later following negative herpes PCR and antibodies in CSF. CSF bacterial PCR and serum quantiferon were also negative. The patient was started on methylprednisolone 1 g/d for 7 days, which was subsequently orally tapered. Initially, there was a slight improvement in muscle strength in the legs (MRC scale grades: 3–4/5 proximally, 5/5 distally). However, 5 days later, the patient developed paraplegia (MRC 1/5), so plasmapheresis was started q.o.d. for 12 days. The interventions resulted in partial radiological resolution of LETM (Figure 2), without any clinical improvement. A repeat CSF analysis showed 7 cells (lymphocytes and monocytes), with normal glucose and no evidence of malignant cells. The patient was subsequently treated with intravenous immunoglobulins (IVIG) 2 g/kg body weight over 5 days, again without any significant improvement. oxcarbazepine 300 mg b.i.d. was added due to tonic spasms, which improved over a few days.

Screening for underlying malignancy by CT thorax and abdomen, serum tumor markers, and a whole body fluorodeoxyglucose positron emission tomography (FDG PET) were negative. Immunological investigations revealed positive antinuclear antibodies (ANA 1/360, Ref. < 1:80), with negative double-stranded DNA (dsDNA) antibodies. Recoverin antibodies in serum and CSF (measured by immunofluorescence) were strongly positive. IL-6 was also increased in the CSF (459 pg/mL), while in serum it was in the normal range (< 5.7 pg/mL). Angiotensin-converting enzyme was also normal in serum (Ref. 20–70 U/L) and CSF (Ref. < 2.5 U/L). The rest of the tested antibodies were negative (Table 1). Due to COVID-19 respiratory infection and urinary tract infection due to *Pseudomonas* and *Candida albicans*, in the course of his hospitalization, which was treated with meropenem and fluconazole, respectively, no further escalation of immunotherapy was decided at the time. Steroids were tapered orally within 1 month. The patient received two additional IVIG courses, on the basis of persistence of positive anti-recoverin antibodies in serum within the next 3 months. Six-month follow-up revealed mild lower limb paresis (MRC 4, proximally more affected than distally) and occasionally urinary and fecal incontinence. The patient was able to walk limited distances with support.

## 3. Discussion

LETM is a serious spinal cord inflammation that spans 3 or more vertebral segments, presenting with motor, sensory, and/or sphincter disorders [4]. LETM can have various autoimmune, inflammatory, or infectious causes; the differential diagnosis comprises myelin oligodendrocyte glycoprotein antibody–associated disease (MOGAD), ADEM, glial fibrillary acidic protein (GFAP) astrocytopathy, multiple sclerosis, systemic lupus erythematosus, sarcoidosis, Sjögren's disease, Behçet's disease, infections (syphilis, herpes viruses, SARS-CoV-2, human immunodeficiency virus (HIV), *Borrelia*, *Mycobacterium tuberculosis*, *Mycoplasma pneumoniae*, and *Streptococcus pneumoniae*), and paraneoplastic, postinfectious, and postvaccination myelitis [4, 5]. Concerning postvaccination myelitis, most cases (over 100) have been reported after SARS-CoV-2 vaccination [6], followed by influenza, human papilloma virus (HPV), hepatitis, measles, rubella, meningococcus, and tetanus vaccination, although individual cases have been reported after other vaccines as well [7]. Myelitis symptoms, being in most cases a combination of motor, sensory, and/or sphincter disturbances of varying severity, occurred commonly one or 2 weeks after vaccination, while a range of disease onset between one day and 1 month has been reported [7, 8]. LETM occurred in almost 70% of cases of myelitides after SARS-CoV-2 vaccination [8]. The vast majority of postvaccination myelitides were treated with at least one immunotherapy such as pulse steroid therapy with or without plasmapheresis and IVIG [7, 8]. Only selected cases received escalation treatment with rituximab [9, 10]. Most patients had partial or full recovery after treatment within a few weeks; death due to complications was very rarely reported [8].

Recoverin is a neuronal calcium–binding protein that is mainly found in the photoreceptor cells of the retina and pineal gland [11]. Anti-recoverin antibodies play an important role in paraneoplastic retinopathy, in which degeneration occurs, mainly in patients with small-cell lung cancer [12]. Anti-recoverin antibodies in neurological syndromes without retinopathy, i.e., with altered consciousness level, status epilepticus, ataxia, parkinsonism, encephalitis with psychiatric symptoms, and polyradiculopathy/plexopathy, have also been reported [13–20]. Two of these cases occurred after SARS-CoV-2 infection [13, 17], and another two occurred after SARS-CoV-2 or influenza vaccination [17, 18]. Anti-recoverin antibodies have also been identified in patients with systemic lupus erythematosus [21]. CSF was found to be inflammatory in a few of those cases [15, 16, 18]. Patients showed variable responses to steroids or IVIG [13–17]. In one case, rituximab was administered, which resulted in neurological improvement and disappearance of anti-recoverin antibodies after administration of three cycles of the drug [14]. The studies reporting the presence of anti-recoverin antibodies in neurological syndromes can be seen in Supporting Table (available here).

The patient's symptoms could be attributed to vaccine-induced multisystem inflammatory syndrome, similar to that reported after COVID-19 vaccination [22]. Although a causal relationship with the vaccine cannot be proven, in favor of this association are the onset of symptoms, which was temporally related to vaccination, the inflammatory CSF, and the increased anti-recoverin antibodies, which further support an abnormal immunological response to the vaccine. The relationship between anti-recoverin antibodies and central nervous system (CNS) involvement is possibly due to the sharing of epitopes between recoverin and CNS endogenous antigens such as calcium-binding recoverin-like proteins [14, 23]. One of the mechanisms of entry of anti-recoverin antibodies into the CNS is the direct route via the bloodstream, influenced by systemic inflammation [23]. Indeed, our patient's first symptoms were those of a systemic inflammation with fever and general weakness. Notably, anti-recoverin antibodies are often detected in the serum after infections such as toxoplasmosis [23]. However, it cannot be excluded that the presence of anti-recoverin antibodies is merely an epiphenomenon. The pathophysiology of neurological involvement associated with anti-recoverin antibodies is yet to be understood, as the protein is only known to be expressed in the retina [13].

Although there is a limitation regarding the causality of anti-recoverin antibodies, RSV vaccination, and myelitis, this association has not been previously reported. The report offers several significant contributions to the literature, i.e., the association of anti-recoverin antibodies with a broader clinical picture than previously recognized, such as myelitis, the association of RSV vaccination with LETM, and lastly, the potential role of anti-recoverin antibodies in vaccine adverse reactions. This case underscores the necessity of performing a widespread search for antibodies in suspected immune-mediated conditions, as the spectrum of antibodies is steadily expanding. By no means, this paper criticizes vaccination in patients, for whom it is indicated, as the risk of a demyelinating or inflammatory CNS disease following vaccination is relatively low and the benefits of vaccinations surpass the potential risks of CNS inflammation [7]. These data also stress the need to explore new ways to improve the safety profile of vaccination techniques. Further future studies with larger sample sizes and longer follow-up after immunotherapy in those vaccine-related immunological conditions are needed to draw firm conclusions on this matter.

## Figures and Tables

**Table 1:** Antibody testing with cell-based assays in the present case.

Antibodies tested in serum and CSF	Result
Antinuclear antibodies (ANA)^∗^	1:360 positive
DNA double-stranded antibodies (DsDNA)^∗^	Negative
Recoverin	Positive
Myelin oligodendrocyte glycoprotein (MOG)	Negative
Aquaporin 4 (AQ4), aquaporin 1 (AQ1)	Negative
Glial fibrillary acidic protein (GFAP)	Negative
N-Methyl-D-aspartate receptor (NMDAR)	Negative
Alpha-amino-3-hydroxy-5-methyl-4-isoxazolepropionic acid receptor (AMPAR)	Negative
GABAB receptor, leucine-rich glioma inactivated 1 (LGI1)	Negative
Contactin-associated protein-like 2 (CASP2)	Negative
Glycine receptor (GlyR)	Negative
Anti-dopamine receptor 2 (DR2)	Negative
Anti-dipeptidyl-peptidase-like protein 6 (DPPX)	Negative
Anti-glutamate receptor (GluR2)	Negative
Immunoglobulin-like cell adhesion molecule 5 (IGLON5)	Negative
Metabotropic glutamate receptor (mGluR1, mGluR5)	Negative
Amphiphysin	Negative
Collapsin response mediator protein 5 (CV2/CRMP5)	Negative
Ma2/Ta, antineuronal nuclear antibody 2 (ANNA2/Ri), Purkinje cell cytoplasmic antibodies type 1 (PCA1/Yo), antineuronal nuclear antibody-type 1 (ANNA1/Hu), anti-Sry-like high mobility group box (SOX1), delta/notch-like epidermal growth factor-related receptor (Tr), zinc-finger protein (Zic4)	Negative

^∗^These antibodies were tested only in serum.

## Data Availability

The data that support the findings of this study are available from the corresponding author upon reasonable request.

## Supporting Information

Supporting Information 1 Supporting Table: Studies reporting the presence of anti-recoverin antibodies in neurological syndromes.

Supporting Information 2 Video legend: Video of the patient at presentation showing tonic spasms of the lower extremities, occurring spontaneously or after a tactile stimulus.
