# Supplementary material for: Longitudinal Extensive Transverse Myelitis After Respiratory Syncytial Virus Vaccination With Positive Anti-Recoverin Antibodies
Source: Case Rep Neurol Med. 2025 Aug 30;2025:6597450. doi: 10.1155/crnm/6597450 (PMC12413940; doi:10.1155/crnm/6597450)
Supplement: Supporting Information 1 — Supporting Table: Studies reporting the presence of anti-recoverin antibodies in neurological syndromes. [file 6597450.f1.docx]

| Authors, year | Gender | Age (years) | Neurological symptoms/signs | MRI findings | CSF findings | EEG findings | retinopathy | Diagnosis of cancer | Presence of auto-antibodies | Treatment | Outcome |
| --- | --- | --- | --- | --- | --- | --- | --- | --- | --- | --- | --- |
| Herzog et al. 2020 [1] | female | 60s | slowly progressive gait disorder, pancerebellar syndrome | brainstem and cerebellum atrophy | normal | n/a | no | no | anti-recoverin (serum) | IVIG, oral steroids  (tapered), subsequently  rituximab  (2 X 375 mg/m^2^) every 12 months for 3 years | subjective improvement after steroids, improvement of ataxia over 3 years |
| Ryu et al. 2020 [2] | male | 65 | ataxia (dysarthria, limb and trunk ataxia, nystagmus), proximal limb weakness | normal | 36 cells/L, normal protein | n/a | yes | small cell lung carcinoma | anti‑Hu, anti-  recoverin (serum) | Methylprednisolone (1 g/d) intravenously  for 5 days | no improvement |
| Avila et al., 2021 [3]  Case 1 | male | 77 | disorientation, sleep-wake cycle  alterations, left IV  nerve palsy,  hyperreflexia, polyradiculopathy or lumbosacral plexopathy | normal | normal | n/a | no | squamous cell carcinoma of the right lung | anti-recoverin,  anti-Ki67,  anti-GAD65 (serum) | IVIG and  steroids | no response |
| Avila et al., 2021 [3]  Case 2 | male | 45 | altered level of consciousness,  psychomotor agitation | normal | normal | desynchronization and generalized  background slowing | no | no | anti-recoverin, anti-titin (serum) | 5-day cycle of IVIG,  methylprednisolone 500 mg/day for 5 days | progressive improvement  after steroids |
| Avila et al., 2021 [3]  Case 3 | male | 82 | focal motor and generalized tonic-clonic status epilepticus | postictal signal  alterations in cortex | normal | n/a | no | bladder tumor | anti-recoverin (serum) | antiepileptic medication | seizures resolved after 48 hours, patient died due to infection |
| Kitazaki et al. 2021 [4] | female | 67 | fever, parkinsonism, somnolence to coma | hyperintense lesions in the basal ganglia bilaterally (DWI, T2, FLAIR) | 30 cells/μL (lymphocytes), no OCBs | generalized slow waves | no | no | anti-recoverin (serum) | prednisolone (15 mg/d) and levodopa 100 mg/d, IVIG for 5 days | parkinsonism persisted, disturbance in  consciousness improved within 2 months. |
| Finsterer et al. 2024 [5] | male | 47 | Headache, concentration problems,  eye movement pain, decreased visual acuity, myoclonus, lower limb weakness | normal brain MRI; nerve MRI: prominent dorsal cervical root  ganglia | elevated protein levels | normal | no | no | anti-recoverin (serum and CSF) | two intravenous methylprednisolone courses,  methotrexate (15 mg/day) and prednisolone orally, immunoadsorption | slight improvement in eye movement, pain and motor function after steroids, no further follow-up |
| Akahane et al. 2024 [6] | female | 25 | executive dysfunction,  disorientation,  upper limb tremor, visual hallucinations, incontinence, agitation, incoherent thoughts, athetoid-like movements, weakness in the lower limbs  suicidal ideation, Cotard and Capgras  delusions | normal | normal | diffuse nonspecific  slowing | no | no | anti-recoverin (serum) | two cycles of IVIG  for 5 days, three cycles of  methylprednisolone 1,000 mg/day for 3 days with oral tapering for 8 months  Fourteen  sessions of bilateral electroconvulsive therapy | improvement of orientation with IVIG and steroids  Delusions markedly improved after electroconvulsive therapy  In remission one year after discharge |
| Vaisvilas et al. 2024 [7]  Case 1 | male | 67 | limbic encephalitis (anterograde amnesia, dementia) | increased signal mesiotemporal bilateral | n/a | focal temporal lobe epilepsy | no | no | anti-recoverin (serum) | immunotherapy (not specified) | no improvement |
| Vaisvilas et al. 2024 [7]  Case 2 | male | 78 | limbic encephalitis (anterograde amnesia) | increased signal mesiotemporal bilateral | n/a | focal temporal lobe epilepsy | no | no | anti-CV2, anti-recoverin (serum) | immunotherapy (not specified) | improvement |

**Abbreviations:** MRI: magnetic resonance imaging, CSF: cerebrospinal fluid, IVIG: intravenous immunoglobulins, OCBs: oligoclonal bands, DWI: Diffusion-weighted imaging, FLAIR: Fluid-Attenuated Inversion Recovery, n/a: not reported

**Supplementary Table.** Studies reporting the presence of anti-recoverin antibodies in neurological syndromes

1. Herzog R, Brüggemann N, Sprenger A, Münte TF: **Recoverin antibody-associated late-onset ataxia without retinopathy**. *BMJ case reports* 2020, **13**(12).

2. Ryu HS, Lee SY, Park DH, Lee JM: **A Case of Paraneoplastic Neurological Syndrome Expressing Dual Antineuronal Antibodies: Anti-Hu and Recoverin**. *Ann Indian Acad Neurol* 2020, **23**(1):133-135.

3. Ávila GM, Escamilla EE, González AP, Corral JAM, Fernández CP, Marcos AR: **Antineuronal antibodies: Anti-recoverin in neurological syndromes without retinopathy. SARS-CoV2 infection as a trigger**. *Neurologia (Engl Ed)* 2022, **37**(5):409-410.

4. Kitazaki Y, Shirafuji N, Takaku N, Yamaguchi T, Enomoto S, Ikawa M, Yamamura O, Nakamoto Y, Hamano T: **Autoimmune basal ganglia encephalitis associated with anti-recoverin antibodies: A case report**. *eNeurologicalSci* 2021, **25**:100382.

5. Finsterer J: **Anti-recoverin Antibody-Associated Post-acute COVID Vaccination Syndrome After BNT162b2 in HLA-B27-Positive Spondylarthritis: A Case Report**. *Cureus* 2024, **16**(8):e66881.

6. Akahane T, Takahashi N, Kobayashi R, Nomura K, Akiho M, Shikama Y, Noto K, Suzuki A: **Case report: A case of anti-recoverin antibody-positive encephalitis exhibiting Cotard and Capgras delusions that was successfully treated with electroconvulsive therapy**. *Front Psychiatry* 2024, **15**:1330745.

7. Vaisvilas M, Petrosian D, Bagdonaite L, Taluntiene V, Kralikiene V, Daugelaviciene N, Neniskyte U, Kaubrys G, Giedraitiene N: **Seroprevalence of neuronal antibodies in diseases mimicking autoimmune encephalitis**. *Scientific reports* 2024, **14**(1):5352.
